# Perinatal Outcomes of Fetuses with Early Growth Restriction, Late Growth Restriction, Small for Gestational Age, and Adequate for Gestational Age

**DOI:** 10.1055/s-0039-1697987

**Published:** 2019-12

**Authors:** Quênya Antunes Silveira Inácio, Edward Araujo Júnior, Luciano Marcondes Machado Nardozza, Caetano Galvão Petrini, Victor Paranaíba Campos, Alberto Borges Peixoto

**Affiliations:** 1 Universidade de Uberaba, Uberaba, MG, Brazil; 2 Escola Paulista de Medicina, Universidade Federal de São Paulo, São Paulo, SP, Brazil; 3 Universidade Municipal de São Caetano do Sul, São Paulo, SP, Brazil; 4 Universidade Federal do Triângulo Mineiro, Uberaba, MG, Brazil; 5 Faculdade de Tecnologia em Saúde, Ribeirão Preto, SP, Brazil; 6 Universidade Barão de Mauá, Ribeirão Preto, SP, Brazil

**Keywords:** fetal growth restriction, small for gestational age, adverse perinatal outcomes, restrição do crescimento fetal, pequeno para a idade gestacional, resultados perinatais adversos

## Abstract

**Objective** To evaluate the association between early-onset fetal growth restriction (FGR), late-onset FGR, small for gestational age (SGA) and adequate for gestational age (AGA) fetuses and adverse perinatal outcomes.

**Methods** This was a retrospective longitudinal study in which 4 groups were evaluated: 1 — early-onset FGR (before 32 weeks) (*n* = 20), 2 — late-onset FGR (at or after 32 weeks) (*n* = 113), 3 — SGA (*n* = 59), 4 — AGA (*n* = 476). The Kaplan-Meier curve was used to compare the time from the diagnosis of FGR to birth. Logistic regression was used to determine the best predictors of adverse perinatal outcomes in fetuses with FGR and SGA.

**Results** A longer time between the diagnosis and birth was observed for AGA than for late FGR fetuses (*p* < 0.001). The model including the type of FGR and the gestational age at birth was significant in predicting the risk of hospitalization in the neonatal intensive care unit (ICU) (*p* < 0.001). The model including only the type of FGR predicted the risk of needing neonatal resuscitation (*p* < 0.001), of respiratory distress (*p* < 0.001), and of birth at < 32, 34, and 37 weeks of gestation, respectively (*p* < 0.001).

**Conclusion** Fetal growth restriction and SGA were associated with adverse perinatal outcomes. The type of FGR at the moment of diagnosis was an independent variable to predict respiratory distress and the need for neonatal resuscitation. The model including both the type of FGR and the gestational age at birth predicted the risk of needing neonatal ICU hospitalization.

## Resumo

**Objetivo** Avaliar o efeito da restrição de crescimento fetal (RCF) precoce, RCF tardio, fetos pequenos constitucionais para idade gestacional (PIG) e fetos adequados para idade gestacional (AIG) sobre resultados adversos perinatais.

**Métodos** Estudo longitudinal e retrospectivo, no qual foram avaliados quatro grupos: 1 — RCF precoce (< 32 semanas) (*n* = 20), 2 — RCF tardio (≥ 32 semanas) (*n* = 113), 3 — PIG (*n* = 59), 4 — AIG (*n* = 476). A curva de Kaplan-Meier foi utilizada para comparar o tempo entre o diagnóstico da RCF e o parto. Regressão logística foi utilizada para determinação dos melhores previsores de resultados perinatais adversos entre os fetos com RCF e PIG.

**Resultados** Os fetos AIGs apresentaram maior tempo entre o diagnóstico e parto, enquanto fetos RCF tardio apresentaram menor tempo (*p* < 0,001). O modelo contendo tanto os tipos de RCF quanto a idade gestacional no momento do parto foi significativo em predizer o risco de internação na unidade de terapia intensiva (UTI) neonatal (*p* < 0,001). O modelo incluindo apenas o tipo de FGR prediz o risco de ressuscitação neonatal (*p* < 0,001), de desconforto respiratório (*p* < 0,001) e de nascimento < 32, 34 e 37 semanas de gestação, respectivamente (*p* < 0,001).

**Conclusão** Os desvios do crescimento, RCF e PIG, foram associados a resultados perinatais adversos. O tipo de RCF no momento do diagnóstico foi variável independente para predizer necessidade de reanimação neonatal e desconforto respiratório. O modelo que incluiu o tipo de FGR e idade gestacional no nascimento prediz o risco de necessitar de internação em UTI neonatal.

## Introduction

Fetal growth restriction (FGR) is influenced by several factors and occurs in ∼ 7 to 15% of all gestations.^1^ ^2^ ^3^ ^4^ Within the same country, it can vary according to cultural and socioeconomic characteristics. The most widely adopted definition of FGR is an estimated fetal weight (EFW) below the 10^th^ percentile for the gestational age.^1^ ^2^ However, some fetuses considered as having FGR do not present pathological growth features and are merely considered as small for gestational age (SGA).^1^ ^2^ ^3^ Small for gestational age differs from FGR, because it includes the majority of constitutionally small, but healthy fetuses with lower risk of adverse perinatal outcome.^4^

The most common cause of FGR is a deficit in the transport of nutrients and oxygen to the fetus through the placenta, but several other maternal factors, such as poor socioeconomic and cultural condition, malnutrition, and chronic vascular disease, as well as fetal factors, such as genetic syndromes and infections, can be involved in this growth impairment.^1^ ^2^ ^3^

Perinatal morbidity and mortality are greater in fetuses with FGR than in normal fetuses, due to more frequent hypoxemia, meconium aspiration, and hypoglycemia.^1^ ^2^ ^3^ Furthermore, FGR is associated with a higher incidence of cardiovascular diseases and diabetes mellitus in childhood and adult life.^5^ ^6^ However, SGA fetuses are also susceptible to adverse perinatal outcomes.^7^ ^8^

The main objective of the present study was to evaluate the adverse perinatal outcomes in early FGR, late FGR, SGA, and adequate for gestational age (AGA) fetuses. The secondary objectives were assessing the time between the diagnosis and the moment of delivery and the main predictors of perinatal adverse outcomes in fetuses with early FGR, late FGR, SGA and AGA.

## Methods

This was a retrospective cohort that evaluated 476 selected pregnant women with singleton fetuses exhibiting adequate growth, and 291 women with singleton fetuses diagnosed with fetal growth impairment. The present study was conducted at the Fetal Medicine Unit of the Mário Palmério Hospital Universitário (MPHU, in the Portuguese acronym) of the Universidade de Uberaba (UNIUBE, in the Portuguese acronym), Uberaba, state of Minas Gerais, Brazil, from August 28, 2013 to November 29, 2016. The cases included in the present study were selected from the Astraia database (Astraia Software GmbH, Munich, Germany). The present study was approved by the UNIUBE Committee of Ethics in Research (CAAE: 99278918.0.0000.5145).

The inclusion criterion was singleton pregnancies with a gestational age between 24 and 41 weeks, calculated from the date of the last menstrual period and confirmed by ultrasound up to 13 weeks and 6 days, who had at least 2 ultrasound examinations between 24 and 41 weeks. Fetuses presenting structural abnormalities or chromosomal diseases diagnosed by ultrasound and confirmed in the postnatal period were excluded, as were births that occurred outside the MPHU and cases whose postnatal data were absent in the database.

Ultrasound examinations were performed by only two examiners (Peixoto A. B. and Petrini C. G.) accredited by the Fetal Medicine Foundation (FMF) and with 8 years of experience in obstetric ultrasonography. All of the examinations were transabdominal and used a Voluson E6 ultrasound system (General Electric Healthcare, Zipf, Austria). The ultrasound examinations followed the protocol of the institution for the evaluation of fetal growth and wellbeing. The following fetal biometric parameters were evaluated: biparietal diameter (BPD), head circumference (HC), abdominal circumference (AC), and femur diaphysis length (FDL), according to the guidelines proposed by the International Society of Ultrasound in Obstetrics and Gynecology (ISUOG).^9^ The estimated fetal weight (EFW) was calculated using the Hadlock formula^10^:

log_10_ [birthweight] = 1.4787 + 0.001837 × BPD^2^ + 0.0458 × AC + 0.158 × FDL − 0.003343 × AC × FDL

In addition to the biometric parameters, the following were also evaluated: largest vertical pocket of amniotic fluid (LVP),^11^ mean uterine artery pulsatility index (PI UtA),^12^ umbilical artery pulsatility index (PI UA),^13^ middle cerebral artery pulsatility index (PI MCA),^14^ middle cerebral artery peak systolic velocity (PSV MCA),^15^ and cerebroplacental ratio (CPR) = PI MCA / PI UA.^16^

The patients were divided into 4 groups: 1 — early-onset FGR, 2 — late-onset FGR, 3 — SGA, 4 — fetuses with appropriate for gestational age (AGA) growth (controls). Appropriate for gestational age was defined if the estimated fetal weight (EFW) was between the 10^th^ and 95^th^ percentile according to the respective gestational age, following normal values of PI UA, PI MCA and mean PI UtA. Fetuses were considered to have early-onset FGR when the gestational age was < 32 weeks and the following criteria were present: EFW or AC below the 3^rd^ percentile for the gestational age or absent end-diastolic flow in UA; EFW or AC below the 10^th^ percentile for the gestational age, associated with a mean PI UtA or PI UA above the 95th percentile for the gestational age. Fetuses were considered to have late-onset FGR when the gestational age was > 32 weeks and the following criteria were present: EFW or AC below the 3^rd^ percentile for the gestational age; EFW or AC below the 10^th^ percentile for the gestational age, associated with a mean PI UA above the 95^th^ percentile for the gestational age, CPR below the 5^th^ percentile for the gestational age, or AC/EFW ratio crossing percentiles > 2 quartiles on growth percentiles.^17^ Fetuses were considered SGA when EFW was between the 3^rd^ and the 10^th^ percentile and the criteria for early- and late-onset FGR diagnosis were not met.

According to our local protocol, 3 ultrasound examinations are recommended, as follows: 11–13 weeks (1^st^ trimester screening for aneuploidies, pre-eclampsia and FGR), 20–24 weeks (anomaly scan), 32–34 weeks (growth scan). However, ultrasound examination can be performed at any time in the presence of obstetrical indication. The ultrasonographic follow-up for SGA and FGR fetuses are individualized according to maternal-fetal conditions. All of the included cases were followed longitudinally with at least two ultrasound examinations during pregnancy, but for analyses, only the parameters measured at the 1^st^ ultrasound examination were used when the FGR diagnosis was made. In the cases of SGA fetuses, in which there was later development of FGR, the parameters of the 1^st^ ultrasound examination with a diagnosis of FGR were used. In cases of AGA fetuses, the 1^st^ ultrasound examination between 24 and 41 weeks was considered for analyses.

The following parameters were considered adverse perinatal outcomes: fetal death, Apgar score < 7 at 5 minutes, hospitalization in a neonatal intensive care unit (ICU), need for neonatal resuscitation, neonatal death within the first 48 hours, birthweight [BW] below the 10th percentile,^18^ hypothermia, hypoglycemia, hypomagnesemia, polycythemia, thrombocytopenia, respiratory distress, and periventricular hemorrhage.

The data were analyzed using IBM SPSS Statistics for Windows, Version 20.0 software (IBM Corp., Armonk, NY, USA). The quantitative variables underwent the Kolmogorov-Smirnov test for normality and were presented as means and standard deviations (SDs). The categorical variables were described as absolute and percentage frequencies and were represented in tables and graphs. The differences between the categorical variables and their proportions were analyzed using the chi-squared test. The effect of FGR on continuous variables was analyzed with the Kruskal-Wallis test. The time elapsed from the diagnosis of FGR until birth was compared using survival analysis through Kaplan-Meier curves. Stepwise logistic regression was used to determine the best predictors of adverse perinatal outcomes in fetuses with some kind of growth impairment in the prenatal period. The odds ratio (OR) for the development of adverse perinatal outcomes with statistical difference between the groups was determined by logistic regression. A receiver operating characteristics (ROC) curve was used to determine the best mean PI UtA value to detect fetuses with weight below the 10^th^ percentile during the prenatal period. The significance level for all tests was *p* < 0.05.

## Results

**Fig. 1:**
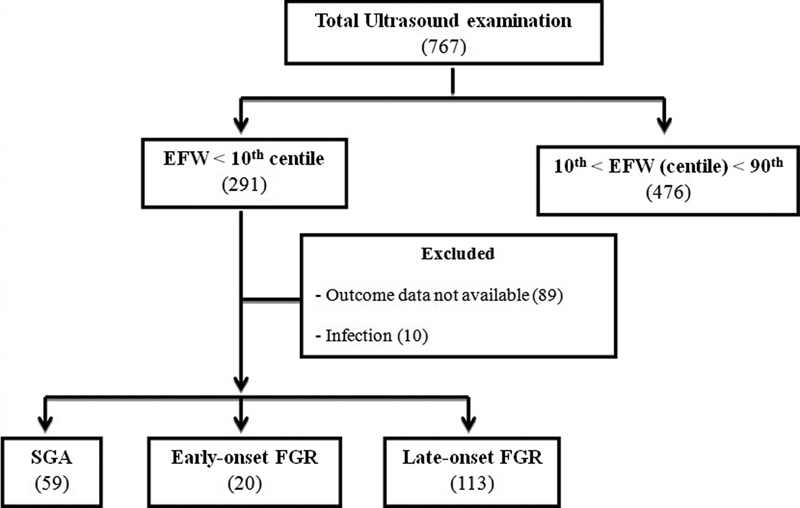
Flowchart with all of the patients enrolled and included in the study.

A total of 767 obstetric ultrasound examinations were evaluated, with gestational ages ranging from 24 weeks to 41 weeks and 4 days. Of this total, 291 examinations (37.94%) had an EFW below the 10^th^ percentile for their gestational age, and 476 (62.05%) had an EFW between the 10^th^ and the 95^th^ percentile. A total of 99 cases were excluded, of which 89 for lacking follow-up, and 10 for infection during pregnancy. Of the 192 remaining cases below the 10^th^ percentile for the gestational age, 67 were SGA. As gestation progressed, 8 SGA fetuses (11.9%) were classified as having late FGR. The final statistical analysis considered 59 SGA fetuses (30.73%), 113 fetuses with late-onset FGR (58.85%), and 20 fetuses with early-onset FGR (10.42%) (Fig. 1).

No statistically significant difference in age, weight, height, or body mass index (BMI) was found between the groups with growth impairment at the moment of diagnosis, even though the gestational age at the moment of diagnosis was considerably lower (31.1 weeks) in patients of the early-onset FGR group than in those with late-onset FGR (36.5 weeks) and than in SGA fetuses (36.3 weeks). However, differences in the number of gestations, parity, gestational age at delivery, time between the diagnosis and delivery, BW, Apgar scores at the 1^st^ and 5^th^ minutes were found to be statistically significant (Table 1).

**Table 1:** Clinical characteristics of the studied population

	AGA (*n* = 476)			Early onset FGR (*n* = 20)			Late onset FGR (*n* = 113)			SGA (*n* = 59)			*p*-value
	Median	Min	Max	Median	Min	Max	Median	Min	Max	Median	Min	Max	
Age (years)	27	14	42	28.5	19	36	23.5	14	40	25.5	16	43	0.265
Weight (kg)	69	40	135	65.7	52	117	62.1	47	102	66.8	42	103	0.063
Height (cm)	162	145	180	163	154	173	162	148	173	160.5	146	176	0.729
BMI (kg/m^2^)	26.2	17.3	46.6	24	19.6	46.3	23.8	18.7	38.9	26	17.5	39.5	0.073
GA at diagnosis (wk)	33.4	25.1	40.1	31.1^d,e^	28.4	32.4	36.5^f^	32	40.7	36.3^a,b^	25.1	39	< 0.001*
Number of pregnancies	2	1	8	1,5	1	3	2^f^	1	6	2	1	7	0.039*
Parity	1	0	5	0,5	0	2	0^f^	0	4	1^b^	0	5	0.01*
GA at delivery (wk)	39	32	41	32^d,e^	28	39	38^f^	32	40	39^a^	32	41	< 0.001*
Diagnosis at delivery (days)	32	0	104	4.0^d^	0	70	4.0^f^	0	52	19.0^b,c^	0	104	< 0.001*
Birthweight (g)	3,250	1,760	4,185	1,512.5^d,e^	765	2,820	2,465^f^	1,380	3495	2807,5^a,b,c^	1485	3830	< 0.001*
Apgar 1^st^ minute	9	4	10	8.5^d,e^	6	9	9	6	10	9^c^	3	10	< 0.001*
Apgar 5t^h^ minute	9	7	10	9^d,e^	7	10	9	8	10	9^a^	7	10	0.004*

Abbreviations: AGA, appropriate for gestational age; BMI, body mass index; FGR, fetal growth restriction; GA, gestational age; Max, maximum; Min, minimum; SGA, small for gestational age; wk, weeks.

* Kruskal-Wallis. Pairwise comparison: a: SGA versus early FGR; b: SGA versus late FGR; c: SGA versus AGA; d: early FGR versus late FGR; e: early FGR versus AGA; f: late FGR versus AGA. Dunn exact test *p* < 0.05. The missing data for the variables age, weight, height, BMI, number of pregnancies, parity, birth weight, Apgar 1^st^ minute, Apgar 5^th^ minute for the AGA, early FGR, late FGR, and SGA groups were: 181, 6, 38, and 15 cases, respectively. The missing data for the variables Apgar 1^st^ minute, Apgar 5^th^ minute for the AGA group were 46 cases. There were no missing data for the following variables: GA at diagnosis, GA at delivery, diagnosis at delivery (days).

With regard to HC measurements, the pairwise comparison showed that fetuses with early-onset FGR had lower median values than all other groups. The same occurred with the median values of AC and FDL. The EFW was also lower in fetuses with early-onset FGR compared with the other groups. Small for gestational age fetuses had higher LVP values than fetuses with both early- and late-onset FGR. Fetuses with early-onset FGR had higher mean PI UtA and PI UA values than the other groups (Table 2).

**Table 2:** Ultrasound characteristics of the studied population at the moment of the diagnosis

	AGA (*n* = 476)			Early onset FGR (*n* = 20)			Late onset FGR (*n* = 113)			SGA (*n* = 59)			*p-value*
	Median	Min	Max	Median	Min	Max	Median	Min	Max	Median	Min	Max	
HC (mm)	301.1	229.7	351.5	264.1^d,e^	224.8	279.4	308.5	271	334.6	307.1^a,b^	199	321.5	< 0.001*
AC (mm)	290.4	198.9	368.2	229.8^d,e^	191	246.5	289.8^f^	241.3	321.1	299.3^a,c^	192.3	322.6	< 0.001*
FDL (mm)	62.1	43.3	76.1	53^d,e^	47.7	58.5	65.3	55.7	73.6	63.4^a,d,c^	40.3	69.5	< 0.001*
HC/AC	1.04	0.9	1.2	1.16^d,e^	0.98	1.31	1.1^f^	0.97	1.2	1^a,b,^	0.93	1.15	< 0.001*
EFW (g)	2,107	727	3,722	1,126.5^d,e^	732	1,425	2,192.5^f^	1,340	2,857	2,281^a,c^	585	2,744	< 0.001*
LVP (cm)	4.9	2.1	9.8	3.7^e^	0	5.4	4.2^f^	0	6.9	4.6^a,b^	1.8	7.3	< 0.001*
UtA PI	0.7	0.42	1.81	1.48^d,e^	0.67	2.58	0.75	0.35	1.71	0.66^a^	0.49	1.67	< 0.001*
UA PI	0.91	0.54	1.47	1.24^d,e^	0.82	4.8	0.96	0.57	1.45	0.9^a^	0.6	1.21	< 0.001*
MCA PI	1.93	0.92	2.8	1.76	1.14	2.36	1.67^f^	0.96	2.6	1.73^b^	1.04	2.66	< 0.001*
Cerebral placental ratio	2.06	1.14	3.92	1.42^e^	0.25	2.73	1.74^f^	0.88	3.42	1.92^a,b^	1.19	3.21	< 0.001*

Abbreviations: AC, abdominal circumference; AGA, appropriate for gestational age; EFW, estimated fetal weight; FDL, femur diaphysis length; FGR, fetal growth restriction; HC, head circumference; LVP, largest vertical pocket; Max, maximum; MCA, middle cerebral artery; Min, minimum; PI, pulsatility index; SGA, small for gestational age; UA, umbilical artery; UtA, uterine artery.

* Kruskal-Wallis. Pairwise comparison: a: SGA versus early FGR; b: SGA versus late FGR; c: SGA versus AGA; d: early FGR versus late FGR; e: Early FGR versus AGA; f: late FGR versus AGA. Dunn exact test *p* < 0.05. There was no missing data for all analyzed ultrasound variables.

**Fig. 2:**
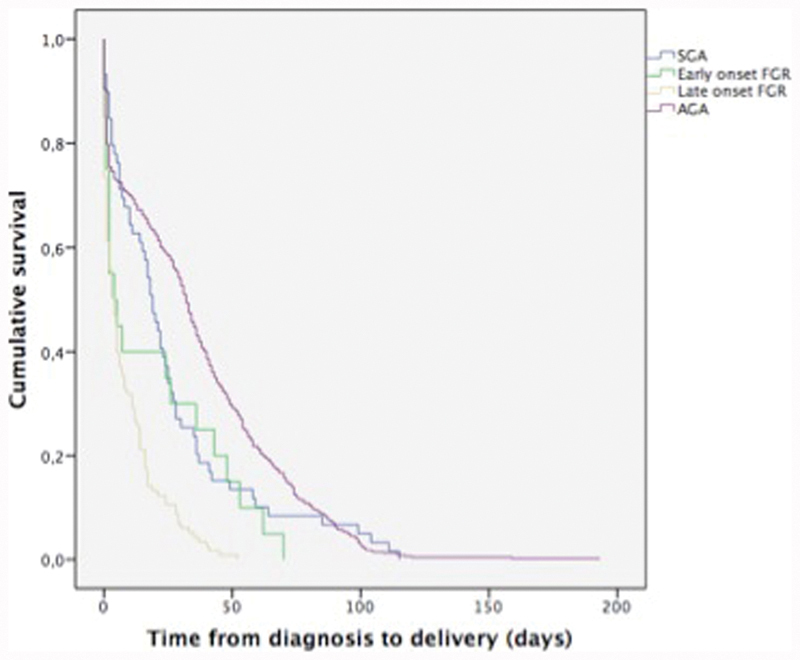
Kaplan–Meier curve including the time elapsed from diagnosis to birth as a function of the type of fetal growth impairment.

A longer maximum elapsed time from the moment of diagnosis to birth was observed for AGA fetuses than for fetuses with late-onset FGR. A statistically significant intergroup difference was observed in the time elapsed from the diagnosis to birth between the initial (Breslow, *p* < 0.001), intermediary (Tarone-Ware, *p* < 0.001), and final (Long Hank, *p* < 0.001) periods of the Kaplan-Meier curve (Fig. 2).

A statistically significant association was found between the types of growth impairment and births before the 32^nd^ (*p* < 0.001), the 34^th^ (*p* < 0.001), and the 37^th^ (*p* < 0.001) weeks of gestation, BW (*p* < 0.001), need for neonatal ICU hospitalization (*p* < 0.001), need for neonatal resuscitation (*p* < 0.001), hypoglycemia (*p* < 0.001), hypomagnesemia (*p* < 0.001), hypothermia (*p* < 0.001), and respiratory distress (*p* < 0.001) (Table 3).

**Table 3:** Adverse perinatal outcomes in fetuses that were appropriate for gestational age and fetuses with intrauterine growth impairment

	AGA			Early onset FGR			Late onset FGR			SGA			*p-value*
	*n*	*n*	%	*n*	*n*	%	*n*	*n*	%	*n*	*n*	%	
**Delivery < 28 wk**													
** Yes**	1	476	0.2	2	20	10.0	0	113	0.0	0	59	0.0	†
** No**	475	476	99.8	18	20	90.0	113	113	100.0	59	59	100.0	
**Delivery < 32 wk**													
** Yes**	6	476	1.3	12	20	60.0	3	113	2.7	2	59	3.4	< 0.001*
** No**	470	476	98.7	8	20	40.0	110	113	97.3	57	59	96.6	
**Delivery < 34 wk**													
** Yes**	13	476	2.7	13	20	65.0	8	113	7.1	3	59	5.1	< 0.001*
** No**	463	476	97.3	7	20	35.0	105	113	92.9	56	59	94.9	
**Delivery < 37 wk**													
** Yes**	39	476	8.2	14	20	70.0	27	113	23.9	12	59	20.3	< 0.001*
** No**	437	476	91.8	6	20	30.0	86	113	76.1	47	59	79.7	
**Birthweight**													
** AGA**	426	472	90.3	5	20	25.0	44	113	38.9	39	59	66.1	< 0.001*
** SGA**	29	472	6.1	15	20	75.0	69	113	61.1	20	59	33.9	
** BGA**	17	472	3.6	0	20	0.0	0	113	0.0	0	59	0.0	
**Apgar < 7 at 5^th^ minute**													
** Yes**	0	430	0.0	0	20	0.0	0	113	0.0	0	59	0.0	†
** No**	430	430	100.0	20	20	100.0	113	113	100.0	59	59	100.0	
**Neonatal ICU**													
** Yes**	20	476	4.2	17	20	85.0	28	113	24.8	6	59	10.2	< 0.001*
** No**	456	476	95.8	3	20	15.0	85	113	75.2	53	59	89.8	
**Fetal demise**													
** Yes**	0	476	0.0	0	20	0.0	0	113	0.0	0	59	0.0	†
** No**	476	476	100.0	20	20	100.0	113	113	100.0	59	59	100.0	
**Neonatal demise**													
** Yes**	0	476	0.0	1	20	5.0	1	113	0.9	0	59	0.0	†
** No**	476	476	100.0	19	20	95.0	112	113	99.1	59	59	100.0	
**Neonatal resuscitation**													
** Yes**	35	476	7.4	14	20	70.0	22	113	19.5	6	59	10.2	< 0.001*
** No**	441	476	92.6	6	20	30.0	91	113	80.5	53	59	89.8	
**Hypoglycemia**													
** Yes**	45	393	11.5	14	20	70.0	45	113	39.8	16	59	27.1	< 0.001*
** No**	348	393	88.5	6	20	30.0	68	113	60.2	43	59	72.9	
**Hypomagnesaemia**													
** Yes**	5	19	26.3	10	20	50.0	9	113	8.0	3	59	5.1	< 0.001*
** No**	14	19	73.7	10	20	50.0	104	113	92.0	56	59	94.9	
**Hypothermia**													
** Yes**	11	474	2.3	13	20	65.0	20	113	17.7	6	59	10.2	< 0.001*
** No**	463	474	97.7	7	20	35.0	93	113	82.3	53	59	89.8	
**Respiratory distress**													
** Yes**	45	475	9.5	17	20	85.0	31	113	27.4	12	59	20.3	< 0.001*
** No**	430	475	90.5	3	20	15.0	82	113	72.6	47	59	79.7	

Abbreviations: AGA, appropriate for gestational age; FGR, fetal growth restriction; ICU, intensive care unit; SGA, small for gestational age; wk, weeks.

* Chi-squared. † It was not possible to perform statistical tests on variables with < 3 patients in any category of answer.

A logistic regression model was created to determine whether the type of FGR and the gestational age at birth are predictors of the need for hospitalization in a neonatal ICU, the need for neonatal resuscitation, and the presence of respiratory distress in comparison with normal fetuses. The model including both the type of FGR and the gestational age at birth was better than the model including only the type of FGR in predicting the risk of needing neonatal ICU hospitalization [x^2^(4) = 286.12; *p* < 0.001; Nagelkerke R^2^ = 0.708], with a 96.6% predictive capability. In contrast, the model including only the type of FGR was better in predicting the risk of needing neonatal resuscitation [x^2^(3) = 42.77; *p* < 0.001; Nagelkerke R^2^ = 0.149] and the risk of presenting respiratory distress [x^2^(3) = 73.80; *p* < 0.001; Nagelkerke R^2^ = 0.180], with predictive capabilities of 89.7% and 86.4%, respectively.

Another logistic regression model was created to determine whether the type of FGR is a predictor of delivery before 32, 34, and 37 weeks of gestation. The model including the type of FGR was a predictor of delivery before 32 [x^2^(3) = 63.7; *p* < 0.001; Nagelkerke R^2^ = 0.708], 34 [x^2^(3) = 59.4; *p* < 0.001; Nagelkerke R^2^ = 0.244], and 37 [x^2^(3) = 57.13; *p* < 0.001; Nagelkerke R^2^ = 0.149] weeks of gestation. The model had predictive capabilities of 97.2%, 95.4%, and 87.4% for the risk of delivery before 32, 34, and 37 weeks, respectively. Table 4 contains the ORs and the CIs for each model tested.

**Table 4:** Risk of adverse events in the neonatal period according to the type of growth impairment and gestational age at birth in comparison with fetuses with adequate growth

	OR	95%CI	*p-value*
**Delivery < 32 wk**			
SGA	2.7	0.54–13.9	0.222
Early FGR	117.5	35.2–391.5	< 0.001*
Late FGR	2.13	0.52–8.7	0.288
**Delivery < 34 wk**			
SGA	1.9	0.52–6.9	0.325
Early FGR	66.1	22.6–193.1	< 0.001*
Late FGR	2.7	1.09–6.7	0.031*
**Delivery < 37 wk**			
SGA	2.9	1.4–5.8	0.004*
Early FGR	26.1	9.5–71.8	< 0.001*
Late FGR	3.5	2.0–6.0	< 0.001*
**Neonatal ICU**			
SGA	2.3	0.59–8.69	0.23
Early FGR	77.1	11.27–527.75	< 0.001*
Late FGR	7.2	2.83–18.01	< 0.001*
**Neonatal resuscitation**			
SGA	1.4	0.573–3.55	0.445
Early FGR	29.4	10.64–81.2	< 0.001*
Late FGR	3	1.7–5.4	< 0.001*
**Respiratory distress**			
SGA	2.4	1.20–4.93	0.013 *
Early FGR	54.14	15.30–191.90	< 0.001*
Late FGR	3.6	2.15–6.04	< 0.001*

Abbreviations: CI, confidence interval; FGR, fetal growth restriction; ICU, intensive care unit; OR, odds ratio; SGA, small for gestational age.

* Binary logistic regression.

A stepwise logistic regression was created to determine if the mean PI UtA, PI UA, PI MCA and CPR (at diagnosis of EFW < 10^th^ centile) are predictors of delivery < 32, 34, and 37 weeks of gestation. Only the mean PI UtA was predictor of preterm delivery < 32 weeks [x2 (1) = 19.0; OR: 9.2; 95%CI: 3.4–24.8; *p* < 0.001; R^2^ Nagelkerke = 0.155]. On the other hand, none of the assessed Doppler parameters were predictors of preterm delivery < 34 and < 37 weeks of gestation.

**Fig. 3:**
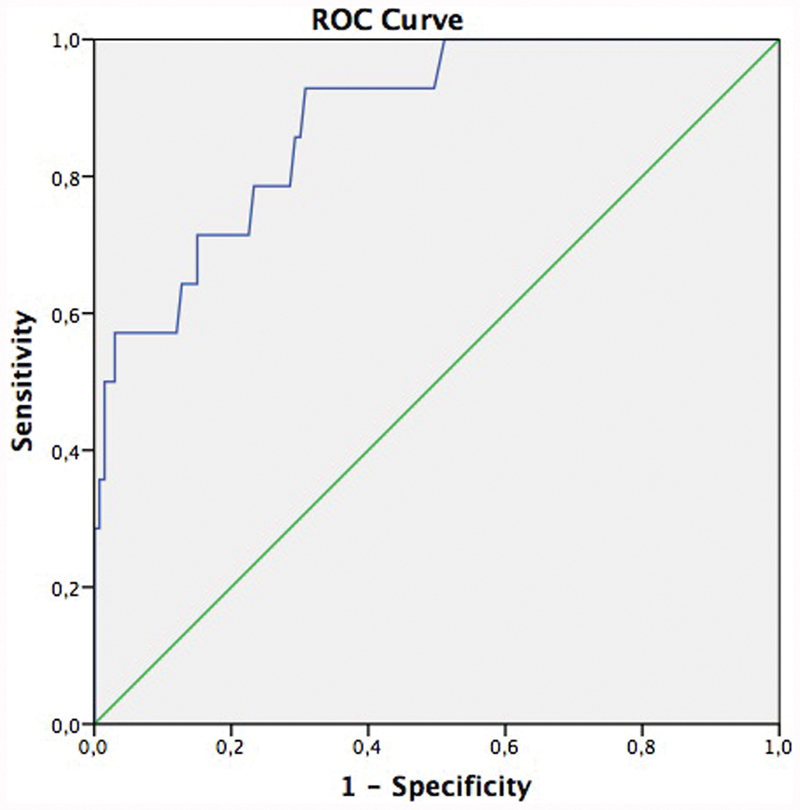
Receiver operating characteristics (ROC) curve to determine the best value for the mean uterine artery pulsatility index (PI UtA) to predict delivery before 32 weeks of gestation in small-for-gestational-age (SGA) fetuses and fetuses with fetal growth restriction (FGR). *Area under the curve: 0.881; *p* < 0.001; 95%CI: 0.796–0.966.

A ROC curve was plotted to determine the best sensitivity and the best mean PI UtA cutoff value to predict delivery before 32 weeks (Fig. 3). Mean PI UtA values of 1.23 and 1.15 were respectively able to correctly identify 57.1% and 65.3% of the fetuses born before 32 weeks of gestation, with respective false-positive rates of 10% and 15%.

## Discussion

Fetal growth restriction affects ∼ 10% of pregnancies, and it is one of the leading contributors to perinatal morbidity and mortality. Its severity greatly influences the risk of adverse perinatal outcomes.^19^ ^20^

Fetal growth restriction occurs when the fetus does not reach its genetic potential for growth and development as consequence of compromise in placental function.^1^ Although this definition seems simple, at the moment, there is no gold standard for the diagnosis of FGR.^4^

In 2016, specialists established a consensus to define, classify, and diagnose FGR using the Delphi procedure.^17^ This consensus was a very important attempt to standardize the diagnosis of FGR, but there is lack of studies to validate these criteria as good predictors of adverse perinatal outcomes. Our study used it for the diagnosis of FGR, which classified fetuses with EFW below the 10^th^ percentile in 3 groups: SGA, early-onset FGR, and late-onset FGR.^17^

The present study aimed to compare perinatal outcomes of SGA, early-onset FGR, and late-onset FGR with AGA fetuses. We observed that fetuses classified as early-onset FGR had the highest risk for adverse perinatal outcomes (need for hospitalization in neonatal ICU, need for neonatal resuscitation, and presence of respiratory distress) compared with AGA fetuses (OR = 77.1, 95%CI:11.27–527.75; OR = 29.4; 95%CI:10.64–81.2; OR = 54.14, 95%CI: 15.30–191.90, respectively).

The fetuses classified as late-onset FGR also had significantly adverse perinatal outcomes compared with AGA fetuses (OR = 7.2, 95%CI:2.8–18.01; OR = 3.0, 95%CI:1.7–5.4; OR = 3.6, 95%CI:2.15–6.04 of need for hospitalization in neonatal ICU, need for neonatal resuscitation, and presence of respiratory distress, respectively). However, fetuses classified with SGA did not show increased risk of need for hospitalization in neonatal ICU or need for neonatal resuscitation (*p* = 0.23 and *p* = 0.44, respectively), but showed increased risk of respiratory distress compared with AGA fetuses (OR = 2.4; 95%CI: 1.20–4.93).

We also found that other adverse perinatal outcomes had a significant correlation between intrauterine growth impairment and AGA groups, like hypothermia (early-onset FGR = 65%, late-onset FGR = 17.7%, SGA = 10.2%, and AGA = 2.3%, *p* < 0,001) and hypoglycemia (early-onset FGR = 70%, late-onset FGR = 39.8%, SGA = 27.1%, and AGA = 11.5%, *p* < 0.001).

Our findings are consistent with previous studies that correlated FGR with adverse perinatal outcomes,^7^ ^19^ ^20^ ^21^ ^22^ ^23^ but the classification of FGR in studies used different criteria. Our study was the first one that used the criteria established by the Delphi procedure.

Regarding fetal death, neonatal death within the first 48 hours and Apgar score < 7 at 5 minutes, it was not possible to perform statistical tests due to the small number of cases of the variables observed in all of the groups

An important factor considered to be an independent determinant of adverse perinatal outcomes is EFW at the moment of diagnosis.^21^ The lower the EFW, the higher the risk of mortality, as attested by a study of 355 pregnant women who were diagnosed with FGR in the 2^nd^ trimester of pregnancy. In that study, the stillbirth rate was significantly higher in patients with EFW below the 5^th^ percentile than that in those with EFW between the 5^th^ and the 10^th^ percentile.^19^ Figueras et al^24^ consider that among fetuses with EFW below the 10^th^ percentile, those below the 3^rd^ percentile present a much higher risk of adverse perinatal outcomes, regardless of the uterine artery Doppler indexes and CPR. In the present study, it was possible to observe that fetuses with early-onset FGR had a lower EFW than all other analyzed groups (*p* < 0.001). In addition, the early-onset FGR group had 75% of the fetuses with low birthweight, the late-onset FGR group had 61.1%, and the SGA group had 33.9%, while the AGA group had only 6.1%. In the study of Fernandez-Rodriguez et al,^25^ only the cases of prenatal FGR that confirmed birth weight below the 3^rd^ percentile by postnatal weight chart had increased risk of adverse perinatal outcomes.

Women with fetuses classified as early-onset FGR were more likely to delivery in earlier gestational ages (median: 32 weeks) compared with other groups: late-onset FGR (38 weeks), SGA (39 weeks) and AGA (39 weeks), *p* < 0.001. However, both groups of FGR (early- and late-onset) showed increased risk for preterm delivery < 34 weeks of gestation (OR = 66.1, 95%CI: 22.6–193.1; OR = 2.7, 95%CI = 1.09–6.7, respectively). The Doppler parameter that had the best predictive value for delivery < 32 weeks was UtA Doppler, with sensitivity of 57.1% for false positive rate of 10% for a PI value of 1.23 (*p* < 0.001). The prematurity in these cases is mainly due to elective deliveries, as observed in the study by Temming et al,^19^ in which FGR in the early second trimester was associated with increased risk of elective deliveries < 37 and < 28 weeks compared with AGA fetuses.

Figueras et al^24^ consider that the only measurement that provides diagnostic and prognostic information for the management of fetuses with FGR is the umbilical artery Doppler examination, either alone or in combination with CPR. They also state that early-onset FGR is highly correlated to severe placental insufficiency and chronic fetal hypoxia, which explains the high proportion of cases with abnormal umbilical-artery Doppler. In contrast, in cases of late-onset FGR, the degree of placental involvement is mild, which explains the normal umbilical-artery Doppler examination in nearly all of the cases.^24^ In our study, we observed the highest median values of mean PI UtA in the early-onset FGR group (1.24 versus 0.96, 0.90, and 0.91 for early-onset FGR, late-onset FGR, SGA, and AGA, respectively, *p* < 0.001), confirming that this group is correlated with severe placental impairment. The median PI of MCA was also significantly lower for the group of late-onset FGR in relation to the SGA and AGA groups (*p* < 0.001), which reflects the importance of this parameter for the diagnosis of late-onset FGR in the differentiation of SGA fetuses, since MCA Doppler has important value for the prediction of adverse outcome among late-onset FGR, whereas UA Doppler is commonly normal in these fetuses.^26^ ^27^

As for the time elapsed (in days) from the diagnosis of growth impairment to birth, a statistically significant correlation was observed between fetuses with late-onset FGR and SGA fetuses, with fetuses with late-onset FGR having lower median values. This is probably due to the fact that late-onset FGR is diagnosed at later gestational ages; therefore, there is less time to interrupt the pregnancy. Moreover, fetuses with late-onset FGR have fewer hemodynamic mechanisms of adaptation to the intrauterine environment compared with fetuses with early-onset FGR, which would explain the shorter interval between the diagnosis and the interruption of pregnancy.^28^ Small for gestational age fetuses can be compared with AGA fetuses, and it can take longer until the pregnancy is interrupted. According to Figueras et al,^24^ gestations of SGA fetuses, after infectious and genetic causes are excluded, may progress up to the 40^th^ week. In gestations of fetuses with late-onset FGR, the moment of interruption varies according to the stage of growth restriction of the fetus, which is determined by the changes observed on ultrasound and Doppler, but interruption of the pregnancy is indicated already at 37 weeks.^24^

The limitations of the present study were the exclusion of 30% of the cases due to the unavailability of outcome, and the retrospective nature of the study. The strengths were the inclusion of fetuses which were classified as FGR according to a recent consensus definition in a single center.^17^

## Conclusion

In summary, we have observed that the criteria established for FGR by the Delphi procedure were good predictors for adverse perinatal outcomes. Additionally, the type of FGR is an independent predictor of neonatal resuscitation and respiratory distress, and when adjusted for gestational age, it becomes also a predictor of the need for neonatal ICU hospitalization, although fetuses classified as SGA are more likely to be constitutionally small and not present placental pathology. These fetuses presented more risk of respiratory distress compared with AGA fetuses. In addition, in our casuistic, 11.8% of the fetuses previously classified as SGA were reclassified as late-onset FGR in their follow-up. Thus, even fetuses classified as SGA need attention to fetal wellbeing and proper follow-up.
